# Normalization of Four Different Types of Pulmonary Hypertension After Atrial Septal Defect Closure

**DOI:** 10.3389/fcvm.2022.876755

**Published:** 2022-06-10

**Authors:** Jana Rubáčková Popelová, Jakub Tomek, Markéta Tomková, Renata Živná

**Affiliations:** ^1^Department of Cardiac Surgery, Na Homolce Hospital, Prague, Czechia; ^2^Pediatric Heart Centre, Motol University Hospital, Prague, Czechia; ^3^Department of Pharmacology, School of Medicine, University of California, Davis, Davis, CA, United States; ^4^Department of Biochemistry and Molecular Medicine, School of Medicine, University of California, Davis, Davis, CA, United States

**Keywords:** pulmonary hypertension, atrial septal defect, hemodynamic type of pulmonary hypertension, normalization, reversibility, mortality

## Abstract

Pulmonary hypertension (PH) is an established risk factor in patients with atrial septal defect (ASD), and its persistence after ASD closure is associated with increased mortality. Therefore, predictors for PH normalization after defect closure are needed. Multiple hemodynamic types of PH exist, but little is known about their prevalence and prognostic value for PH normalization after ASD closure. We carried out a retrospective study on 97 patients (76% female, median age at ASD closure 58 years) with four types of PH determined predominantly by right heart catheterization: hyperkinetic, pulmonary arterial hypertension, isolated post-capillary, and combined pre- and post-capillary. We investigated the frequency of the PH types and their prognostic significance for PH normalization after ASD closure. Frequency of PH types before ASD closure in our study was: hyperkinetic 55%, pulmonary arterial hypertension 10%, isolated post-capillary PH 24%, and combined PH 11%. Hyperkinetic PH type was positively associated with PH normalization after ASD closure (78% patients normalized), remaining a significant independent predictor when adjusted for age at closure, sex, heart failure, and NYHA. Hyperkinetic PH patients also had significantly better survival prognosis versus patients with other PH types (*p* = 0.04). Combined PH was negatively associated with PH normalization, with no patients normalizing. Pulmonary arterial hypertension and isolated post-capillary PH had intermediate rates of normalization (60 and 52%, respectively). In summary, all four hemodynamic types of PH are found in adult patients with ASD, and they can be used to stratify patients by their likelihood of PH normalization and survival after ASD closure.

## Introduction

Pulmonary hypertension (PH) represents an important risk factor associated with reduced functional capacity and increased mortality in patients with atrial septal defect (ASD) (1–6). Moreover, persistence of PH after ASD closure is strongly associated with increased mortality (4, 7, 8). On the other hand, patients with normalization of PH after ASD closure have similar outcome as patients without PH (7). The decision of whether to close ASD in patients with PH presents a complex clinical dilemma (9, 10). Therefore, it is highly important to predict in which patients with ASD and PH the defect closure will lead to PH normalization and in which patients the closure may be detrimental with right heart failure and persistence or even progression of PH.

The guidelines give limits for safe defect closure for pulmonary vascular resistance (PVR) <3 Wood Units (WU) or 4 WU × m^2^ and contraindication of defect closure for PVR more than 5 WU or 8 WU × m^2^ (2, 11, 12). However, the guidelines do not specify the probability of PH normalization after defect closure, an important factor for survival (4, 7, 8). Moreover, many studies (including guidelines) deal only with pulmonary arterial hypertension (PAH) in congenital shunt lesions (2, 3, 13, 14), although it is just one of four hemodynamic types of PH in adults with ASD (9, 15). We hypothesized that the hemodynamic types of PH may be predictive of normalization of PH following ASD closure.

Existing ESC/ERS guidelines for the diagnosis and treatment of PH recognize pre-capillary PH, isolated post-capillary PH (IpcPH), and combined pre- and post-capillary PH (CpcPH) (2, 15). Pre-capillary PH involves pulmonary arterial hypertension (PAH), defined by elevated pulmonary vascular resistance (PVR ≥ 3 WU). One of the causes of PAH can be congenital heart disease (CHD) with shunt (2). Although most studies on ASD with PH focus just on PAH (3, 14, 16), hyperkinetic PH (H-PH), characterized by normal or only modestly increased PVR (<3 WU) and normal pulmonary capillary wedge pressure (PCW ≤ 15 mmHg), is another type of PH described in ASD (9, 17, 18). Interestingly, hyperkinetic PH is currently not discussed in ESC/ERS guidelines for the diagnosis and treatment of pulmonary hypertension (2, 15). IpcPH is characterized by increased left atrial pressure or PCW (>15 mmHg), which can in some cases worsen after defect closure (10, 19). Little is known about CpcPH in patients with ASD.

The objective of this study was to characterize the frequency of the four types of PH (H-PH, PAH, IpcPH, and CpcPH) as determined predominantly by right heart catheterization (RHC) in adults with ASD, to assess their prognostic value for predicting PH normalization after defect closure, and to evaluate patient mortality in the four PH types.

## Methods

### Patients

Following institutional ethics committee approval (Na Homolce Hospital), we performed a retrospective observational study including all the adult patients in our database with the diagnosis of ASD (type secundum, sinus venosus or coronary sinus defect) with PH who underwent defect closure, with known PH type before defect closure. Patients with incomplete atrioventricular septal defects (ASD type primum) or ASD combined with other hemodynamically important CHD were excluded. PH was defined for the purpose of this study as mean pulmonary arterial pressure (mPAP) ≥ 25 mmHg (2). The contraindications for ASD closure were: PVR > 5 WU not responding to advanced therapy or (previously) to acute vasodilation testing which is not recommended any more (11, 12). Another contraindication for ASD closure was increase in left atrial mean pressure during temporary balloon occlusion > 10 mmHg compared to baseline in patients with postcapillary PH (19). Mortality data were obtained from the national mortality register. Processing of human data was carried out in accordance with institutional guidelines.

### Catheterization

During right heart catheterization (RHC) the right atrial pressure, sPAP, mPAP, pulmonary capillary wedge pressure (PCW), and left atrial pressure were measured. Cardiac output (pulmonary flow) was measured by Fick method preferentially with measured oxygen consumption, but also with estimated oxygen consumption or dye-dilution or thermodilution, according to the cath-lab facilities. PVR was calculated, and the shunt was quantified by oximetry. Left heart catheterization with coronary angiography was performed according to the usual criteria. Re-catheterization after defect closure was performed in the case of suspected moderate or severe PH from echocardiography.

For the purpose of this study, the four investigated hemodynamic types of PH were defined as specified in Table 1 (2, 15, 17, 18). IpcPH and CpcPH were distinguished by PVR, not by diastolic pressure gradient (15).

**Table 1:** Definition of hemodynamic types of PH in ASD.

	**mPAP (mmHg)**	**PCW (mmHg)**	**PVR (WU)**
Hyperkinetic (H-PH)	≥ 25	≤ 15	<3
Pulmonary arterial hypertension (PAH)	≥ 25	≤ 15	≥3
Isolated post-capillary PH (IpcPH)	≥ 25	>15	<3
Combined pre- and post-capillary PH (CpcPH)	≥ 25	>15	≥3

*mPAP, mean pulmonary arterial pressure; PCW, pulmonary capillary wedge pressure; PVR, pulmonary vascular resistance*.

### Echocardiography

The size of the ASD was assessed by transesophageal echocardiography. In addition to RHC-determined hemodynamic types of PH, 19 out of the 97 patients (all 19 with H-PH) were diagnosed by echocardiography (20–22). These patients had only mild PH (mPAP 25–30 mHg), with no signs of left heart disease, high pulmonary flow and near-normal PVR (assessed by echocardiographic method which has good correlation with invasive PVR) (21). Therefore, we consider the diagnosis of H-PH in these patients as reliable. All other 78 patients had diagnosis of the type of PH assessed by RHC.

PH normalization was assessed by echocardiography using the method of mPAP assessment described by Aduen et al. (22). Mean PAP = mean pressure difference between right ventricle and right atrium, which is derived from the velocity-time integral (VTI) of the tricuspid regurgitation and the estimated right atrial pressure (RAP) is added. This method correlates closely with invasive measurements (22). Only in the rare case of absence of tricuspid regurgitation we used the alternative method of peak Doppler velocity of the pulmonary regurgitation, mPAP = 4V^2^ + RAP. Both methods are recommended in a review article by Parasurman (20). When PH was suspected after defect closure, RHC was used for the diagnostics. PH was considered normalized when mPAP was <25 mmHg (2).

### Statistical Methods

**Figure 1:**
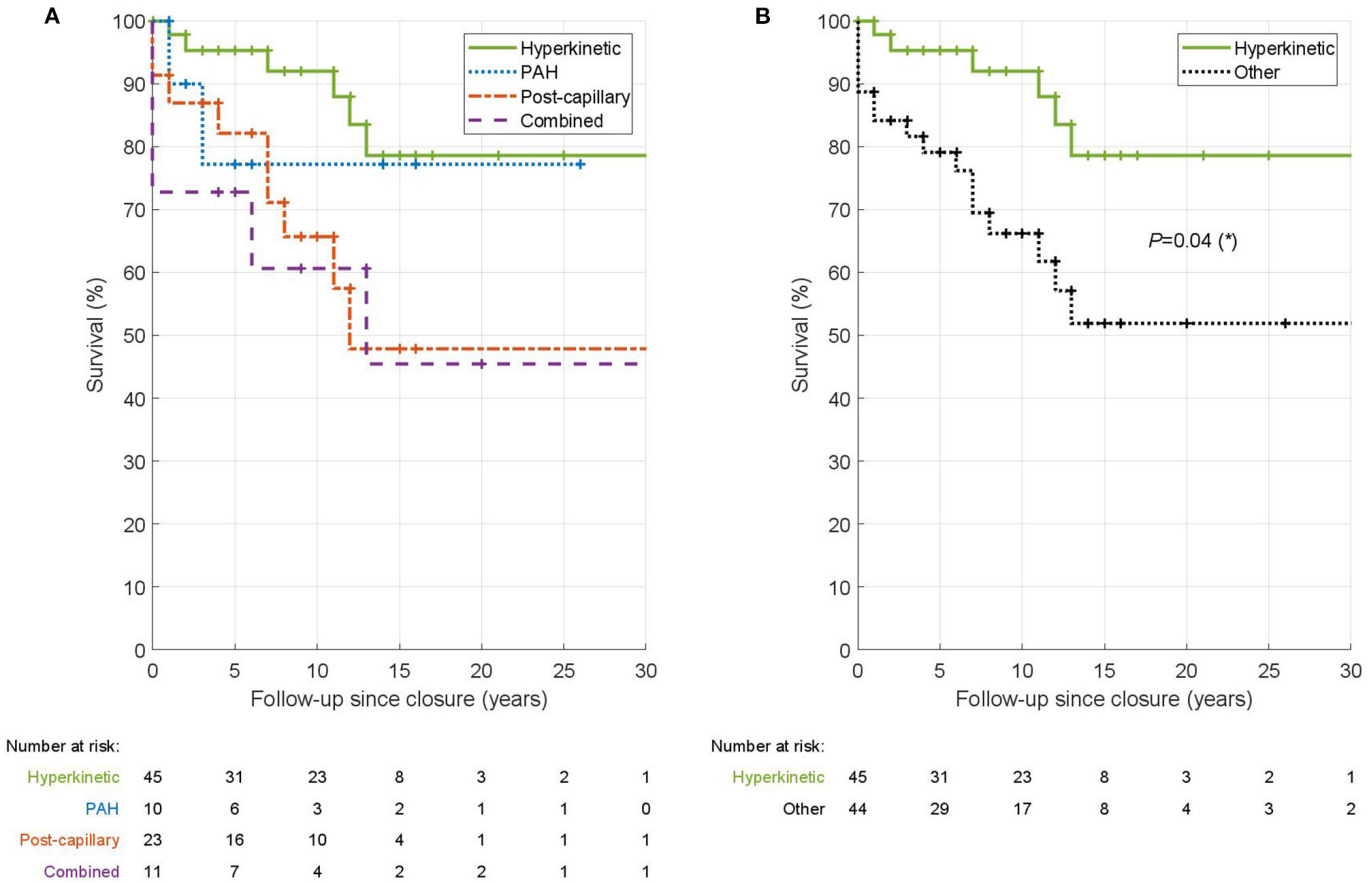
Kaplan-Meier survival analysis of hemodynamic types of PH in ASD. In the left panel **(A)** is shown the comparison of survival among the four hemodynamic types. In the right panel **(B)** is the comparison of H-PH patients vs. patients with other PH types pooled together. The tables underneath the Kaplan-Meier curves give the number of patients who were alive and followed at the given time point. The log-rank test was used to test the significance of survival difference between hyperkinetic patients and all other patients.

Kruskal-Wallis ANOVA was used to compare clinical features between PH types (Table 2). Cox proportional-hazards ratio was used to study the association between clinical features and PH normalization (Table 3). One exception within Table 3 is the CpcPH, where the zero rate of normalization precludes the use of the Cox proportional-hazards ratio, and a Fisher test was used instead on the underlying contingency table to obtain the *p*-value. We verified that all covariates in the Cox model fulfill the proportional hazards assumption, using the MATLAB fitcox function, which is based on the scaled Schoenfeld residuals, as derived by Grambsch and Therneau (23). The variables significant in univariable Cox proportional hazard ratio model and sex and age at closure and the NYHA class were then included in a multivariable model. Kaplan-Meier survival analysis was used to compare survival rates in the four PH types (Figure 1). Wilcoxon rank-sum test was used to assess the difference in NYHA in PH-normalized vs. PH-persisting patients. Only patients with data available on normalization were included in the Cox proportional-hazards and Kaplan-Meier analyses. Hypothesis testing was two-sided and *p* < 0.05 was considered statistically significant. Data were analyzed using MATLAB (R2021b).

**Table 2:** Cohort summary.

**Clinical variable**	**H-PH (*n* = 53)**	**PAH (*n* = 10)**	**IpcPH (*n* = 23)**	**CpcPH (*n* = 11)**	**All (*n* = 97)**	***p*-value**
PH normalization	78% (35/45)	60% (6/10)	52% (12/23)	0% (0/11)	60% (53/89)	4.8·10^−5^
Sex (female)	77% (41/53)	90% (9/10)	57% (13/23)	100% (11/11)	76% (74/97)	0.025
Age at diagnosis	47.0 [34.0–59.0] (*n* = 53)	59.0 [37.0–60.0] (*n* = 10)	50.0 [10.0–65.0] (*n* = 23)	66.0 [52.8–72.0] (*n* = 11)	50.0 [28.0–61.0] (*n* = 97)	0.18
(years)						
Age at closure	52.0 [42.0–61.3] (*n* =53)	59.0 [51.0–65.0] (*n* =10)	60.0 [51.0–70.8] (*n* =23)	69.0 [58.0–74.0] (*n* =11)	58.0 [46.8–65.0] (*n* =97)	0.009
(years)						
NYHA before	2.0 [2.0–3.0] (*n* =53)	3.0 [2.0–3.5] (*n* =10)	3.0 [2.6–3.0] (*n* =23)	3.0 [2.6–3.0] (*n* =11)	2.5 [2.0–3.0] (*n* =97)	2.4·10^−5^
closure						
ASD size (mm)	20.0 [16.5–25.5] (*n* =48)	28.0 [20.0–39.3] (*n* =7)	15.5 [11.0–21.0] (*n* =20)	19.5 [14.0–22.5] (*n* =8)	20.0 [15.0–24.8] (*n* =83)	0.06
Qp/Qs	2.4 [1.8–3.0] (*n* =48)	2.0 [1.7–2.5] (*n* =9)	2.2 [1.6–2.6] (*n* =18)	2.5 [2.2–2.7] (*n* =6)	2.3 [1.8–3.0] (*n* =81)	0.63
HF before closure	13% (7/53)	40% (4/10)	35% (8/23)	55% (6/11)	26% (25/97)	0.011
mPAP before	30.0 [27.0–33.8] (*n* =51)	32.0 [28.0–40.0] (*n* =10)	33.5 [28.0–44.0] (*n* =22)	37.5 [35.0–45.0] (*n* =10)	32.0 [28.0–36.3] (*n* =93)	0.009
closure (mmHg)						
sPAP before	44.0 [39.8–50.0] (*n* =53)	47.5 [45.0–63.0] (*n* =10)	50.0 [42.0–59.8] (*n* =23)	55.0 [52.0–69.5] (*n* =11)	47.0 [41.0–55.0] (*n* =97)	0.0006
closure (mmHg)						
Surgical closure	77% (41/53)	100% (10/10)	100% (23/23)	64% (7/11)	84% (81/97)	0.012
PH follow-up	14.5 [9.0–48.5] (*n* =44)	14.0 [10.0–40.0] (*n* =10)	31.0 [12.0–60.8] (*n* =23)	13.0 [4.0–84.3] (*n* =11)	16.0 [10.0–51.0] (*n* =88)	0.74
length (months)						

*Binary variables are given as percentage (positive/all cases in the PH type). Numerical variables are given as median [interquartile range] (n), where n is the number of patients in the PH group with available data. Qp/Qs, pulmonary-to-systemic flow; HF, heart failure; sPAP, systolic PAP; mPAP, mean PAP; H-PH, hyperkinetic PH; PAH, pulmonary arterial hypertension; IpcPH, Isolated post-capillary PH; CpcPH, combined pre- and post-capillary PH. “Surgical closure” corresponds to sternotomy, minithoracotomy, or robotic thoracoscopy (in the other cases, transcatheter closure was used)*.

**Table 3:** Univariable and multivariable Cox proportional-hazards analysis for association of PH normalization and clinical features.

**Univariable Cox proportional-hazards models**				
**Feature**	**PH normalized (*****n*** **=** **53)**	**PH persisting (*****n*** **=** **36)**	**Hazard ratio [CI]**	***p*** **-value**
Sex (female)	81% (43/53)	69% (25/36)	1.633 [0.819–3.258]	0.16
Age at diagnosis (years)	49.0 [33.5–60.0] (*n* =53)	57.5 [18.0–66.0] (*n* =36)	1.008 [0.996–1.021]	0.19
Age at closure (years)	53.0 [42.0–62.3] (*n* =53)	60.5 [54.5–69.5] (*n* =36)	1.003 [0.984–1.022]	0.74
NYHA before closure	2.0 [2.0–3.0] (*n* =53)	3.0 [2.0–3.0] (*n* =36)	0.685 [0.449–1.047]	0.08
ASD size	20.0 [14.3–24.8] (*n* =47)	20.0 [16.3–23.3] (*n* =29)	1.003 [0.969–1.038]	0.86
Qp/Qs	2.2 [1.8–3.0] (*n* =44)	2.3 [1.9–3.5] (*n* =29)	0.895 [0.637–1.255]	0.52
HF before closure	17% (9/53)	42% (15/36)	0.456 [0.221–0.942]	0.034
H-PH	66% (35/53)	28% (10/36)	2.414 [1.344–4.338]	0.003
PAH	11% (6/53)	11% (4/36)	1.236 [0.526–2.904]	0.63
IpcPH	23% (12/53)	31% (11/36)	0.610 [0.313–1.188]	0.15
CpcPH	0% (0/53)	31% (11/36)	N/A	1.6·10^−5^
mPAP before closure (mm Hg)	30.0 [27.0–33.8] (*n* =51)	35.0 [29.0–40.0] (*n* =34)	0.991 [0.955–1.029]	0.65
sPAP before closure (mm Hg)	45.0 [40.0–52.3] (*n* =53)	50.0 [45.0–61.5] (*n* =36)	0.995 [0.971–1.019]	0.67
Surgical closure	85% (45/53)	78% (28/36)	1.230 [0.576–2.628]	0.5924
**Multivariable Cox proportional-hazards model**				
**Model**	**Feature**	**Hazard ratio [CI]**	**p-value**	
H- PH adjusted for age at closure, sex, HF, and NYHA	Hyperkinetic PH	2.37 [1.22–4.6]	0.01	
	Age at closure (years)	1.0 [0.99–1.03]	0.56	
	Sex	1.43 [0.7–2.94]	0.33	
	HF before closure	0.53 [0.23–1.22]	0.13	
	NYHA before closure	1.12 [0.64–1.96]	0.68	

*Binary variables are given as percentage (positive/all cases). Numerical variables are given as median [interquartile range] (n). Distinct PH types were represented as four separate binary variables (1=presence of the PH type). Hazard ratios (HR) are expressed with regards to PH normalization, i.e., hazard ratio > 1 means a positive association between the feature and PH normalization. Qp/Qs, pulmonary-to-systemic flow; HF, heart failure; sPAP, systolic PAP; mPAP, mean PAP; H-PH, hyperkinetic PH; PAH, pulmonary arterial hypertension; IpcPH, Isolated post-capillary PH; CpcPH, combined pre- and post-capillary PH. In the case of CpcHP, the Cox proportional-hazards analysis cannot be applied given the zero normalization rate; the Fisher-test was used to calculate the p-value from the underlying contingency table instead*.

## Results

A total of 97 adult patients after ASD closure (70% *via* sternotomy, 8% *via* minithoracotomy, 5% *via* robotic thoracoscopy, 17% *via* transcatheter) were included in the study. The median age at the ASD closure was 58 years [47–65 IQR] and 76% of the patients were female. Before defect closure, 53 (55%) patients had H-PH, 10 (10%) PAH, 23 (24%) IpcPH, and 11 (11%) CpcPH. During long-term follow-up for PH normalization after ASD closure (median 16 months, IQR 10–51), PH normalized in 53 patients out of 89 (60%) for whom the data on normalization were available.

PH normalization differed significantly between the hemodynamic types (*p* = 4.8·10^−5^, Table 2). Patients with H-PH manifested the greatest rate of normalization (78%), while normalization was lowest in patients with CpcPH (0%). Other features significantly different between hemodynamic types were: sex, age at closure, NYHA class before closure, presence of heart failure (HF) before closure, the measurements of sPAP (systolic PAP) and mPAP (mean PAP) before closure, and the proportion of surgical closure (as opposed to transcatheter closure) (Table 2).

Three clinical variables were significantly predictive of PH normalization (Table 3, top). While H-PH type was positively predictive of PH normalization (*p* = 0.003, HR = 2.4 [1.34–4.34]), CpcPH type was negatively predictive (*p* = 1.6·10^−5^). Presence of HF before closure was negatively predictive of normalization [*p* = 0.034, HR = 0.46 (0.22–0.94)]. In addition, the NYHA class was borderline nonsignificantly negatively predictive of PH normalization [*p* = 0.08; HR = 0.69 (0.45–1.05)].

Next, we used a multivariable Cox proportional-hazards model for the H-PH, further accounting for age at closure, sex, heart failure, and the NYHA class. H-PH remained a significant independent predictor of PH normalization (*p* = 0.01) (Table 3, bottom). Given the zero normalization rate in the CpcPH group, a similar analysis could not be carried out for this hemodynamic type.

In addition, we compared the improvement in NYHA class after ASD closure between PH-normalized and PH-persisting patients. The PH-normalized patients showed a significantly increased improvement in NYHA class compared to PH-persisting patients (mean improvement of 0.85 vs. 0.28; *p* = 0.007).

Finally, we compared survival of patients in the four hemodynamic types using Kaplan-Meier survival analysis (Figure 1A). H-PH type showed the best survival (*p* = 0.04; logrank test compared to other PH types) with a 10-year survival of 92% (Figure 1B). PAH and IpcPH types had intermediate 10-year survival (77 and 66%, respectively). The survival was lowest in CpcPH type with a 10-year survival of 61%.

Due to the exclusion criterion PVR > 5 WU in our study, advanced therapy (bosentan and sildenafil) was used in 7 (7%) of our ASD patients only (6 after defect closure, one before and after closure). Majority of patients with advanced therapy (5 out of 7) did not normalize PH (3x CpcPH, 1x PAH, 1x H-PH). The patient with H-PH had PVR 2.7 WU before closure and developed CpcPH after ASD closure with PVR 5 WU and died due to heart failure with severe left ventricular dysfunction 12 years after ASD closure.

## Discussion

This study is the first to evaluate the frequency of the four different types of PH in ASD patients, as well as their predictive value for PH normalization and mortality. We observed that the PH types are highly predictive of PH normalization after ASD closure, with H-PH being a significant positive predictor, even after adjusting for age, sex, NYHA class, and presence of heart failure. Conversely, CpcPH was a significant negative predictor of PH normalization. Moreover, we observed a substantially higher improvement in NYHA class after ASD closure in PH-normalized patients compared to PH-persisting patients. Our study therefore shows the importance of assessing the hemodynamic PH type for the risk stratification and design of treatment strategy of ASD patients. The RHC is recommended for PH type diagnosis; the echocardiography-based studies cannot usually conclusively determine the hemodynamic type of PH, particularly in more severe PH.

The reversibility of PH is known to be affected by the severity of pulmonary vascular disease (PVD) and the extent of remodeling of the pulmonary vasculature (14). In the past, the prediction of PH normalization was assessed by histology from open lung biopsies; however, it was abandoned for risk of the procedure and non-uniform distribution of histological changes. More recently, non-invasive predictors of PH normalization have been suggested, reporting age at closure, NYHA class, degree of tricuspid regurgitation, and baseline PAP as significant predictors (4, 16, 24).

Interestingly, women comprised the vast majority of patients with PVR ≥ 3 WU (PAH and CpcPH, Table 2) in our study, although there was no significant difference in the rate of PH normalization between men and women. On the contrary, the hazard ratio for PH normalization was non-significantly better for women (HR = 1.6; *p* = 0.16), (Table 3).

### Hyperkinetic PH

Hyperkinetic PH (H-PH) in ASD is a consequence of increased pulmonary blood flow due to the left-to-right shunt on the atrial level. Volume overload of the highly distensible pulmonary vascular bed in young patients may or may not lead to increased PAP. If the increased pulmonary pressure is proportional to the increased pulmonary flow, the PVR is low with no pulmonary vascular disease (PVD). With time, excess pulmonary blood flow can lead to early PVD changes with marked remodeling of the distal pulmonary vasculature and loss of the elastic properties and stiffening of the large proximal pulmonary arteries. High pulmonary flow leads to upregulation of flow-sensitive genes with endothelial cell dysfunction and neomuscularization, which is reversible when the high pulmonary flow is normalized (13). In the case of progression of pulmonary vascular bed remodeling, PVR increases. If PVR exceeds 3 WU, the H-PH turns to PAH, suggesting the presence of a significant PVD. It is worth mentioning that the cut-off of 3 WU is relatively arbitrary, and PVR > 2 WU could be also considered abnormal (15). The impact of mildly increased PVR (2.5–3 WU) on patient prognosis in H-PH remains to be assessed.

In our study, H-PH was the most frequent PH type (55% patients). It was an independent positive predictor of PH normalization after ASD closure, manifesting the greatest normalization rate of 78%. Patients with H-PH had also significantly better long-term survival after defect closure compared to other types of PH, with a 10-year survival of 92%. This is in line with the mild PVD in H-PH, as well as high pulmonary pressure proportional to high pulmonary flow, resulting in normal or mildly increased PVR.

More attention therefore needs to be paid to the hyperkinetic type of PH in ASD. Some patients with high PAP due to H-PH may be erroneously considered inoperable by inexperienced cardiologists, even though their prognosis after ASD closure might have been very good with PH normalization. In addition, hyperkinetic PH patients misdiagnosed as having PAH may be prescribed expensive advanced therapy with no evidence of its utility for hyperkinetic PH. Therefore, consultation in specialized expert centers including RHC is important for patients with signs of moderate or severe PH according to echocardiography.

### Pulmonary Arterial Hypertension

While being the most studied PH type in connection with shunt CHD, PAH was the least frequent type in our study (present in 10% patients with ASD). It showed 60% PH normalization rate and 77% 10-year survival rate after ASD closure. PAH is characterized by increased PVR (≥ 3 WU) and more severe PVD (2, 15). This includes not only the medial hypertrophy and intimal hyperplasia but also resistance to apoptosis, neointimal fibrosis with vascular lumen occlusion, and plexiform lesions (13). While high likelihood of irreversible changes in pulmonary vascular bed can be expected in patients with PVR above 5 WU (2, 11, 12, 25), such patients are contraindicated for ASD closure and therefore not a part of this study. However, more precise prediction of PH normalization in PAH patients with PVR of 3–5 WU is needed. The role of new methods such as nuclear imaging, circulating biomarkers, or specific genetic mutations should be evaluated in the future (13). Advanced pulmonary vasodilator therapy, before and/or after ASD closure, may be helpful in stabilization of the clinical state with NYHA improvement and lowering of PAP and PVR, even if not leading to complete PH normalization. Advanced therapy may lower PVR below 5 WU in some patients and thus allow ASD closure, usually with fenestration (treat-and-repair strategy) if significant left-to right shunt is still present (Qp:Qs > 1.5) (12).

### Isolated Post-capillary PH

IpcPH was the second most frequent PH type (24% patients) with 52% normalization rate and 66% 10-year survival rate since ASD closure. IpcPH is a consequence of left heart failure with increased filling pressures and PCW ≥ 15 mmHg. The reason may be the co-incidence of ASD with mitral regurgitation or stenosis or with left ventricular (LV) systolic dysfunction. IpcPH may be also a consequence of LV diastolic dysfunction resulting from altered geometry of the LV due to severely dilated right ventricle. In post-capillary PH, the defect serves as a pop-off valve alleviating the risk of pulmonary edema in the case of high left atrial pressure. It is therefore important to exclude further increase of filling pressures after defect closure. Based on literature and our experience, temporary occlusion testing, fenestrated closure, heart failure treatment before defect closure and correction of valvular heart disease and arrhythmias are important and recommended (7, 19, 26).

### Combined Pre- and Post-capillary PH

Little is known about clinical and physiological characteristics of CpcPH generally (27) and in connection with ASD. In our study, CpcPH was present in 11% patients with ASD. It was a significant negative predictor of PH normalization in our study (no patients normalized) and resulted in the lowest 10-year survival of 61%. Patients with this PH type also had the highest mPAP and sPAP and the highest rate of heart failure before ASD closure (55%). Patients with ASD and CpcPH were the oldest (median age of 66 and 69 years at diagnosis and defect closure, respectively). Patients with CpcPH develop more severe PVD than patients with IpcPH and resemble PAH in hemodynamic and genetic characteristics (27). Design of an optimal treatment strategy in these patients needs further research. In our experience, measures should be applied beyond ASD closure (if PVR is below 5 WU), such as heart failure treatment, concomitant valvular or antiarrhythmic surgery, fenestrated closure or pulmonary vasodilator treatment in individual cases and individual dosage before and/or after defect closure (27).

Patients with ASD and IpcPH or CpcPH due to left heart disease (mitral valve disease, left ventricular dysfunction, etc.) are sometimes excluded from research studies concerning ASD (14, 16). Consequently, information on their prognosis and treatment strategies is lacking. Our results demonstrate substantial differences between normalization rates and prognosis in IpcPH (52% normalized) vs. CpcPH (0% normalized), highlighting these as two functionally highly distinct types.

### Summary

In conclusion, we investigated four different hemodynamic types of PH based on RHC, which may accompany ASD in adulthood. They differed in frequency within the studied cohort, likelihood of normalization following ASD closure, and in mortality. The high frequency and strong prognostic value of hyperkinetic PH suggests that this PH type is worth including and discussing in future ESC/ERS guidelines (2, 15). The knowledge of the PH type in ASD can help to guide a tailored treatment strategy.

## Limitations of The Study

We used an older cut-off value for the diagnosis of PH with mPAP ≥ 25 mmHg in our study, based on the guidelines from the time of data collection and study design (2). According to our experience, inclusion of patients with mPAP 20–24 mmHg would only increase the number of patients with mild hyperkinetic PH who did not have RHC.

Given the retrospective nature of the study, certain clinical variables are missing in some of the patients. However, Tables 2, 3 contain the patient counts for each comparison so that the number of available measurements is clear.

Due to the exclusion criterion PVR > 5 WU in this study, advanced therapy was used only in 7 of our ASD patients with PH, which does not allow detailed analysis.

## Data Availability Statement

The raw data supporting the conclusions of this article will be made available by the authors, without undue reservation.

## Ethics Statement

The studies involving human participants were reviewed and approved by Ethical Committee of the Na Homolce hospital. Written informed consent for participation was not required for this study in accordance with the national legislation and the institutional requirements.

## Author Contributions

JR conceived the study, collected the patient data, and is the guarantor of the study. RŽ contributed to data collection. JT and MT carried out data analysis. JT and JR wrote the initial draft, with all authors subsequently carrying out critical revisions. All authors contributed to the article and approved the submitted version.

## Funding

This work was supported by Ministry of Health, Czech Republic–conceptual development of research organization, Nemocnice Na Homolce–NNH, Prague, Czech Republic, 00023884, IG160201 and Motol University Hospital, Prague, Czech Republic 00064203, IG6004.

## Conflict of Interest

The authors declare that the research was conducted in the absence of any commercial or financial relationships that could be construed as a potential conflict of interest.

## Publisher's Note

All claims expressed in this article are solely those of the authors and do not necessarily represent those of their affiliated organizations, or those of the publisher, the editors and the reviewers. Any product that may be evaluated in this article, or claim that may be made by its manufacturer, is not guaranteed or endorsed by the publisher.
